# Unveiling CD59-Antibody Interactions to Design Paratope-Mimicking Peptides for Complement Modulation

**DOI:** 10.3390/ijms24108561

**Published:** 2023-05-10

**Authors:** Annamaria Sandomenico, Alessia Ruggiero, Emanuela Iaccarino, Angela Oliver, Flavia Squeglia, Miguel Moreira, Luciana Esposito, Menotti Ruvo, Rita Berisio

**Affiliations:** Institute of Biostructures and Bioimaging (IBB), National Research Council (CNR), I-80131 Napoli, Italy; annamaria.sandomenico@cnr.it (A.S.); alessia.ruggiero@cnr.it (A.R.); emanuela.iaccarino@gmail.com (E.I.); oliver.angelaa08@gmail.com (A.O.); flavia.squeglia@cnr.it (F.S.); luciana.esposito@cnr.it (L.E.); menotti.ruvo@cnr.it (M.R.)

**Keywords:** protein structure, complement, peptide, paratope, epitope, anti-viral, infection, cancer

## Abstract

CD59 is an abundant immuno-regulatory human protein that protects cells from damage by inhibiting the complement system. CD59 inhibits the assembly of the Membrane Attack Complex (MAC), the bactericidal pore-forming toxin of the innate immune system. In addition, several pathogenic viruses, including HIV-1, escape complement-mediated virolysis by incorporating this complement inhibitor in their own viral envelope. This makes human pathogenic viruses, such as HIV-1, not neutralised by the complement in human fluids. CD59 is also overexpressed in several cancer cells to resist the complement attack. Consistent with its importance as a therapeutical target, CD59-targeting antibodies have been proven to be successful in hindering HIV-1 growth and counteracting the effect of complement inhibition by specific cancer cells. In this work, we make use of bioinformatics and computational tools to identify CD59 interactions with blocking antibodies and to describe molecular details of the paratope–epitope interface. Based on this information, we design and produce paratope-mimicking bicyclic peptides able to target CD59. Our results set the basis for the development of antibody-mimicking small molecules targeting CD59 with potential therapeutic interest as complement activators.

## 1. Introduction

The complement system is one of the major players in the humoral innate immune response [1]. In humans, it assumes a wide range of functions, such as synapse maturation, clearance of immune complexes, bacterial killing, cancer containment, tissue regeneration and lipid metabolism [2,3,4,5]. Additionally, the complement system represents an important bridge between the innate and adaptive immune responses, as it regulates and activates adaptive immune response, and can be activated by antibodies from the adaptive immune system [5]. Consistently, complement dysregulation is involved in severe pathogenesis, such as atypical hemolytic uremic syndrome, C3 glomerulopathy and paroxysmal nocturnal hemoglobinuria [6].

The complement system is composed by a complex and intricate network of over twenty humoral proteins and membrane-bound glycoproteins. It can be activated via three different pathways, noted as classical, lectin and alternative. All pathways induce the activation of the C3 convertases that through the cleavage of C3 generate the large fragment C3b that can subsequently yield the opsonins iC3b and C3d [5], and the smaller fragment C3a that promotes inflammation and regulates further additional metabolic responses [2]. The activated C3b can further initiate the lytic pathway by contributing to the formation of the C5 convertase and the assembly of the Membrane Attack Complex (MAC). The formation of MAC in the outer membrane of some bacteria can induce their lysis. The activation of C5 releases C5a, which is a potent anaphylatoxin that can attract macrophages and neutrophils, as well as activate mast cells [5]. Complement activity is normally regulated through the dissociation dynamics of the C3 and C5 convertases and the assembly of MAC. The formation of the convertases is regulated by a series of soluble and cell surface-anchored regulators of complement activation (RCAs).

Of these, CD55 (or DAF—decay-accelerating factor), factor H, factor I, MCP and CR1 regulate C3 convertases, whereas CD55 and factor H regulate C5 convertases [1,7]. The regulation of the assembly of the MAC complex is mainly operated by the RCA CD59 [8]. CD59, such as CD55, is a glycoprotein that attaches to membranes of virtually all nucleated cells through GPI (Glycosylphosphatidylinositol) anchors, although it also exists in a soluble form that can be found in several tissues and urine. Recent cryo EM studies have shown that CD59 binds the complement proteins C8 and C9 at the membrane to prevent the insertion and polymerisation of MAC pores [8]. The inhibitory action of CD59 in the terminal activation of the complement system makes it an attractive target for diseases or conditions where the assembly of MAC could be beneficial for the host. It has been shown that neoplastic cells overexpress both CD59 and CD55 to resist complement-mediated cytolysis (CMC) [9]. Consistently, the use of bispecific antibodies targeting these RCAs and the targets of conventional cancer immunotherapy regimens, such as trastuzumab (anti-HER2) and rituximab (anti-CD20), enhance the complement-dependent cytotoxicity (CDC) against breast cancer and non-Hodgkin’s leukemia cells, respectively [10,11,12,13,14]. Importantly, HIV-1 virions are vulnerable to complement-mediated lysis (virolysis). Indeed, plasma from HIV-1-infected individuals mediates virolysis [15]. However, HIV-1 can incorporate host CD55 and CD59, thus counteracting virolysis [16]. It has been recently shown that targeting CD55 and CD59 using monoclonal antibodies induces CDC at the surface of HIV-1-infected lymphocytes and enhances virolysis [17]. The knowledge of key interactions occurring between these RCAs and their specific antibodies would allow the design of smaller antibody mimicking molecules, to take advantage of their enhanced pharmacokinetic properties and reduced costs of production. However, structures of complexes between CD59 and its specific antibody are hitherto unknown. Antibody mimicking bicyclic peptides were previously developed using phage display [18,19]. Here, we adopt molecular modelling and dynamics to describe such interactions between CD59 and a neutralising monoclonal antibody to rationally design peptide-based bicyclic molecules mimicking the antibody paratope region. The molecules have been prepared by chemical synthesis and studied by biophysical tools to assess their ability to bind CD59.

## 2. Results

### 2.1. Modelling of the Blocking Minibody MB59 and Identification of Paratope and Epitope Regions

**Figure 1:**
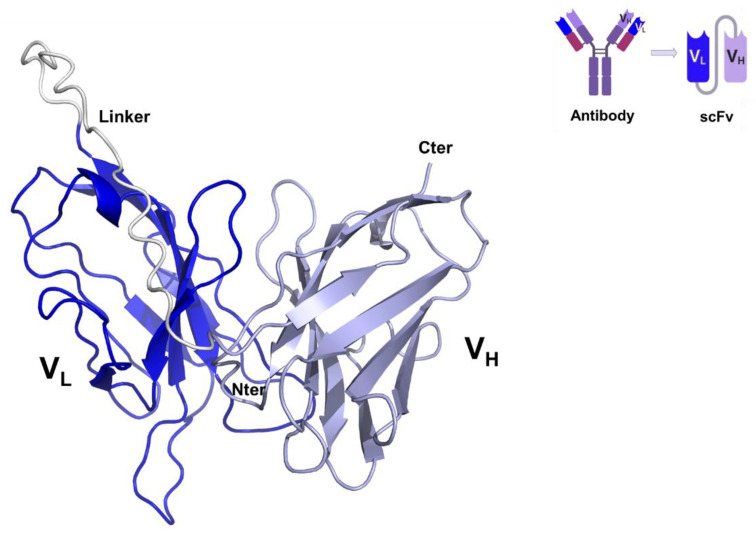
Cartoon representation of the CD59—blocking scFv, MB59. V_L_ and V_H_ chains are drawn in blue and lilac purple, respectively. The inset reports a scheme of scFv chain organisation.

A single-chain variable fragment (scFv), named minibody MB59, has been previously shown to efficiently block the complement inhibitory action of CD59 [20]. Using the MB59 sequence (US patent US8034902B2), we built a homology model of MB59. The crystal structure of the single chain omalizumab scFv (PDB entry 6tcs, 65% sequence identity) was selected as the most suitable template using the software HHPRED [21] and a consensus-based sequence alignment against sequences deposited in the PDB. Therefore, a highly reliable homology model of MB59 was built using the software MODELLER [22] (Figure 1). In parallel, modelling of MB59 using Artificial Intelligence (AlphaFold2.0 software) [23] produced a nearly superposable model, with root means square deviations (RMSD) on backbone atoms of 1.0 Å, and the main differences are located in the flexible link region between V_H_ and V_L_ chains (residues 115–135). The stereochemical quality of the MB59 3D model was improved by energy minimisation with GROMACS [24] and used to identify the paratope regions of MB59 in the interaction with CD59.

**Figure 2:**
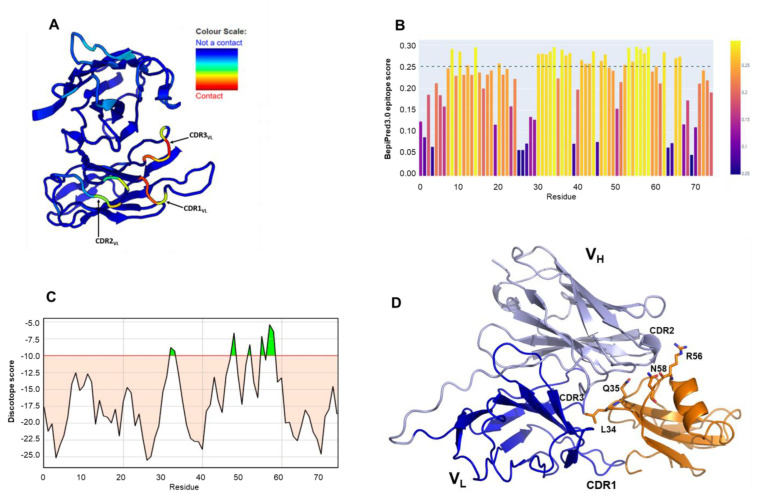
Paratope and antigen predictions. (**A**) Paratope propensity of MB59 residues. Cartoon representation of MB59 model highlighting the scores of the paratope propensity prediction analysis. Colour scheme spans from red (highest paratope propensity score) to blue (lowest paratope propensity score). (**B**) CD59 sequence-based epitope prediction using BepiPred3.0; lightest yellow corresponds the highest scores. (**C**) Structure-based discontinuous epitope prediction using DiscoTope. Green peaks correspond to highest scores, over the threshold (-10). (**D**) Cartoon representation of the MB59-CD59 computed using AlphaFold2.0. High ranking epitope residues are labelled.

Antibody binding to the epitope is mainly formed by the three hypervariable regions termed complementarity-determining regions (CDRs). Using the Antibody i-Patch algorithm implemented in the SAbPred interface [25], we identified the highest paratope propensities for the CDR3 of the V_L_ chain (residues 95–101), followed by the CDR1 loop of the V_L_ (residues 35–39). The CDR2 loop of the V_L_ chain presented the lowest paratope propensity among the three CDRs, indicating a lower contribution in CD59 interaction (Figure 2A). For epitope prediction, we used the sequence-based B-cell epitope predictor tool BepiPred3 [26] which identified the strongest immunogenic surfaces of CD59 in three main regions, embedding residues 9–16, 30–40 and 55–60, with the last two showing the highest scores (Figure 2B). Consistent predictions were obtained using the structure-based tool DiscoTope [27], using the crystal structure of human CD59 (pdb code 2ofs). Maximum scores were computed for residues belonging to the C-terminal end of the sole α helix of CD59 structure (Arg56 and Asn58), followed by residues Leu34 and Gln35 in the central β-sheet (Figure 2C). To further examine MB59-CD59 interactions, we modelled MB59-CD59 complex using AlphaFold2.0 and performed Molecular Dynamics (MD) simulations, using GROMACS (Figure 2D).

### 2.2. Molecular Dynamics Simulations of MB59-CD59 Complex

**Figure 3:**
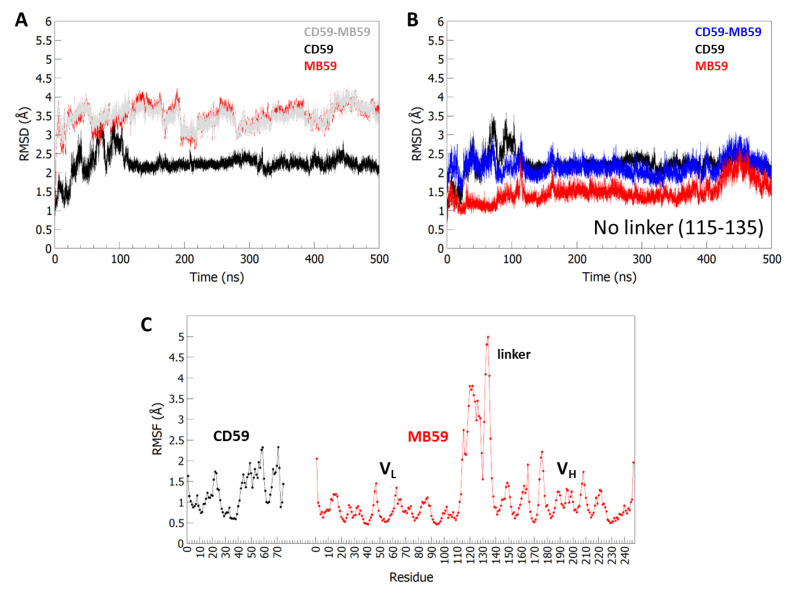
RMSD and RMSF analysis from MD simulations. (**A**) Time evolution of RMSD (Å), computed on Cα atoms, for the MD59-CD59 and isolated chains. In (**B**) RMSD evolution upon removal of the linker 115–135. The colour code is indicated. (**C**) RMSF values, calculated on backbone Cα atoms in the equilibrated region (100–500 ns).

AlphaFold2.0 generated a highly reliable structure model, with Local Distance Difference Test score, pLDDT >85. A 500 ns MD simulation was carried out from this starting model using the Amber99sb all-atom force field with tip3p water model in the isobaric-isothermal (NPT) ensemble at 300 K, using periodic boundary condition (Section 4). The evaluation of root-mean-square deviations (RMSD) (calculated on the protein Cα atoms) between the starting models and the trajectory structures shows limited conformational variations for CD59, with RMSD values lower than 2.5 Å (Figure 3A,B). Higher values are computed for MB59 and for the CD59-MB59 complex (close to 3.5 Å). However, this is to be ascribed to the high conformational flexibility of the linker between the V_L_ and V_H_ chains of MB59 (residues 115–135). When this linker is removed from the calculation, RMSD values computed on Cα atoms of MB59 and CD59-MB59 complex drop close to 1.5 Å and 2.5 Å, respectively (Figure 3B). A low conformational variability of the CD59-MB59 complex is confirmed by root-mean-square fluctuations (RMSF) values, computed in an equilibrated region of trajectory (last 400 ns of the MD simulation). Indeed, high RMSF values characterise the linker region whereas those of the rest of the structure range between 0.5 and 2.5 Å (Figure 3C).

**Figure 4:**
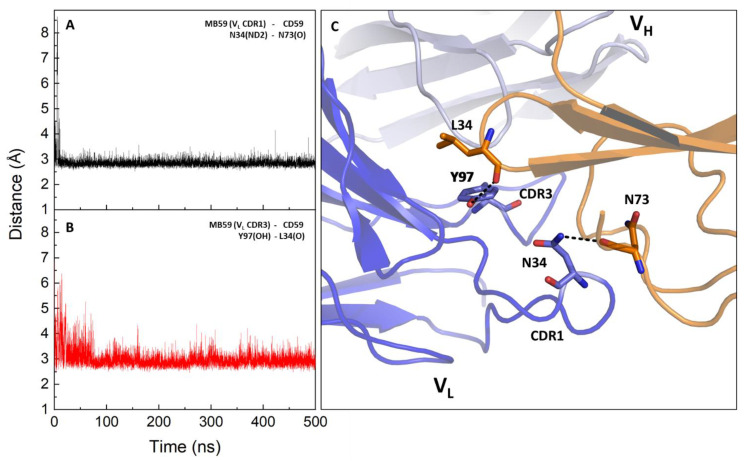
Hydrogen bonding distance evolution of representative key interactions in the MD trajectory of the MB59-CD59 complex. (**A**,**B**) Time evolution of H-bonding interactions involving MB59 CDR1 (**A**) and CDR3 (**B**). (**C**) Cartoon representation showing the H-bonding interactions on a representative structure of the trajectory (the closest to the average structure).

The MD trajectory was deeply analysed to pinpoint the most frequent and stable interactions between CD59 and MB59. By using the MDcons package [28], we computed the map of inter-residue contacts between the MB59 and CD59 chains along the trajectory sampled every 100 ps. In this analysis, two residues are considered to be in contact if at least two heavy atoms are within 5 Å. The map is colour coded considering the conservation of the contact during the simulation (Figure S1). By considering contacts present in more than 70% of frames out of the total analysed trajectory frames, we extracted the most involved regions in the recognition interface of both molecules. As shown in Table 1, all CDR loops of MB59 are to some extent involved in interactions with CD59. However, consistent with our paratope and epitope predictions, the largest number of contacts and the highest frequencies are observed for CDR1 and CDR3 of the V_L_ chain (Figure S1). By scoring interactions based on their percentage of persistence in the trajectory structures (see Methods for selection criteria) and on the average distance between the interacting atoms, we identify fully stable H-bonds detected between the side chain of Asn34 (CDR1, V_L_ chain) of MB59 and the main chain of Asn73 of CD59 and between the side chain of Tyr97 of MB59 and the main chain of Leu34 of CD59 (Table 1, Table 2 and Table 3, Figure 4). Transient hydrogen bonding interactions also exist between CDR1 of the V_H_ chain, with the CD59 loop Leu55-Glu57 (Table 2). Consistently, MB59 V_L_ chain CDR loops also form multiple non-bonded interactions with CD59, with a higher number of interactions involving CDR1 and CDR3 (Table 3, Figure S2).

### 2.3. Design and Preparation of MB59 Paratope Mimicking Peptides

The structural information gathered on interactions between CD59 and MB59 was employed to design paratope-mimicking peptides. We designed three peptides based on modified versions of the CDR1 and CDR3 loops of the MB59 V_L_ chain since they showed the highest paratope forming probability and the most frequent interactions, as determined by molecular dynamics (MD) simulation analysis. To enable rigidification of the CDR loop structures that favours recognition with the target CD59 epitope, peptide sequences were designed in order to accommodate in positions with low paratope propensity prediction and flanking the CDR selected portions (Table 4). These three cysteines strategically allow the conversion of linear peptides to bicyclic molecules around a 1,3,5-tris (bromomethyl)benzene (TBMB) scaffold [18]. The bicyclic peptides were designed to be conformationally sound and mimic the three-dimensional conformation and positioning of the CDR loops of MB59. Linear peptides were prepared by solid-phase stepwise synthesis using standard Fmoc-chemistry protocols on an automated Syro peptide synthesiser. Peptides amidated at the C-terminus and acetylated at the N-terminus were purified by Reverse-Phase High-Performance Liquid Chromatography (RP-HPLC) on a Jupiter C18 column (Section 4) applying a 5% to 70% linear gradient of 0.1% TFA (Trifluoroacetic acid) in acetonitrile (CH_3_CN) over 15 min (15 mL/min flow rate). After bi-cyclisation, peptides were again purified and characterised by LC-ESI-TOF (Figures S3–S5).

### 2.4. Binding Affinity of Paratope-Mimicking Peptides to CD59

**Figure 5:**
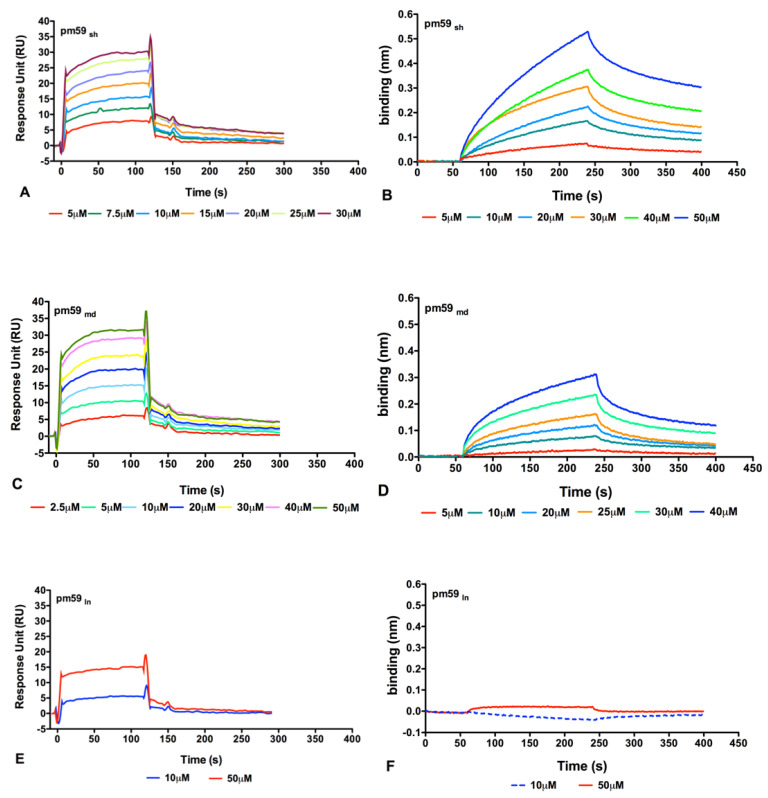
Overlay of sensorgrams obtained for the binding of the three bi-cyclic peptides to CD59 using SPR (**A**,**C**,**E**) and BLI (**B**,**D**,**F**) techniques. The curves of pm59_sh_ are reported in (**A**,**B**), the curves of pm59_md_ are reported in (**C**,**D**) and the curves of pm59_ln_ are reported in (**E**,**F**).

The bicyclic peptides were tested for their ability to bind to recombinant human CD59. Direct binding assays were performed both using Surface Plasmon Resonance (SPR) and Biolayer Interferometry (BLI) label-free techniques (Figure 5). In both cases, after immobilisation of the protein on the sensor chips, the peptides were tested at increasing concentrations between 2.5 µM and 50 µM. As shown in Figure 5, both short (A-B) and medium (C-D) versions of pm59 bicyclic peptides bound CD59 in a dose-dependent manner, showing K_D_ values in the low micromolar range. SPR data fitting, obtained applying a 1:1 Langmuir model, indeed, provided affinity constant (K_D_) values of 2.42 × 10^−6^ M and 3.13 × 10^−6^ M for pm59_sh_ and pm59_md_, respectively. The kinetic parameters of the fitted binding curves are reported in Table 5. Binding experiments were next repeated using BLI to confirm the recognition. Despite the molecules showing a different kinetic profile, as expected for different techniques, the estimated K_D_s obtained using the 1:1 Langmuir fitting model were in the same range, exhibiting values of 9.18 × 10^−6^ M for pm59_sh_ and 4.39 × 10^−6^ M for pm59_md_ (Table 6). No significant binding was measured for the longer version of pm59, pm59_ln_ (Figure 5E,F).

## 3. Discussion

The complement system is one of the cornerstones of the human immune response. Its potential for wreaking havoc in the case of dysregulation demands strong regulatory mechanisms [2,3,4,5]. Indeed, complement overactivation is associated with diverse pathologies such as protein-losing enteropathy, rheumatoid arthritis, systemic lupus erythematosus and ischemia [29,30]. Moreover, it is a distinctive feature of severe SARS-CoV-2 infection [31,32,33]. Repressing the terminal lysis capacity of the complement system through the involvement of the inhibiting RCA protein CD59 is characteristic of cancer resistance and of the action of infectious pathogens, which tend to hijack the complement system regulators to defend from its aggression [10,11,12,13,14]. For instance, CD59 is incorporated in the viral capside, such as HIV and vaccinia, in an effort to avoid complement-mediated lysis [16]. Antibodies targeting CD59 provide a promising therapeutical approach against specific cancer malignancies [10,11,12,13,14] and viral infections [17]. However, due to their molecular size, they exhibit well-recognised unfavourable pharmacokinetics associated with poor tissue penetration [34]. Therefore, we have sought alternative agents able to modulate the CD59 activity. Paratope-mimicking molecules were previously developed using phage display [18,19]. In this framework, we have developed a protocol to rationally design and synthesise paratope-mimicking peptides based on structural data and combining computational and experimental approaches [7,35]. In this work, we use paratope and epitope prediction tools, AI and MD simulations to design paratope-mimicking peptides (pm59) able to bind CD59. The synthesised peptides embed portions of the mini-antibody MB59, comprising CDR1 and CDR3 loops of the V_L_ domain, that we identified by MD simulations as those responsible for the most stable and persistent inter-molecular interactions with CD59. Binding affinities of these peptides to CD59, measured by SPR and BLI, are all in the low µM range, suggesting that the design approach is accurate, and that this strategy can be successfully adopted for the generation of active molecules with drastically reduced size compared to bulky molecules such as antibodies. Moreover, bicyclic peptides have greater conformational rigidity and metabolic stability than linear and monocyclic peptides [36]. Being chemically synthesised, they allow for site-directed chemical modifications, such as chemical modifications to enhance binding affinity and pegylation to enhance metabolic stability.

Although we are aware of the fact that pm59 peptides need further functional experiments and optimisation, our results underline the feasibility and relevance of engineering paratope-mimicking molecules with potential applications as theragnostic agents in diseases associated with CD59 overexpression. Indeed, they guarantee the advantage of low-cost structure-guided site-directed chemical modifications. Finally, human CD59 shares a high sequence identity with those of primates, close to 90% in gorillas and several scimpanzé and orangutan species. This property is open to applications in veterinary medicine.

## 4. Materials and Methods

### 4.1. Modelling Studies

Homology modelling of MB59 was obtained after a consensus-based sequence alignment against all sequences in the Protein Databank using the software HHPRED. This procedure identified the crystal structure of the single chain omalizumab scFv (PDB entry 6tcs 65% sequence identity) as a suitable template for modelling. The homology model was built with MODELLER [22] and the stereochemical quality of the models built was improved by energy minimisation using GROMACS [24]. MB59 was also modelled using the Colab server (https://colab.research.google.com/github/sokrypton/ColabFold/blob/main/AlphaFold2.ipynb, accessed on 1 March 2023), a slightly simplified version of AlphaFold v2.0 [23]. The reliability of the AF predictions was assessed both by the Local Distance Difference Test (LDDT) score and by the Predicted Aligned Error (PAE) score. The same software was used to model the MB59-CD59 complex structure. Structure superpositions were performed using the DALI software. All structures were analysed and displayed using PyMOL.

### 4.2. Paratope and Epitope Prediction

To predict the paratope region of MB59, we used the Antibody i-Patch algorithm implemented in the SabPred interface [25]. Starting models for the paratope prediction were the structure of the homology model of CD59-blocking minibody obtained here and the deposited structure of CD59 (PDB code 2ofs). In SabPred, each residue is scored according to its probability to be involved in antigen binding, upon comparison with previously described antibody–antigen structures [25]. The prediction of the residues of the epitope of CD59 was performed using BepiPred-3.0 [26], a sequence-based tool and the software Discotope [27], which provides structure-based predictions of discontinuous epitopes.

### 4.3. Molecular Dynamics

The GROMACS package [24] was used to perform a 500 ns MD simulation of MB59-CD59 complex using previously established protocols [37] by applying the Amber-ff99SB all-atoms force field [38] and the TIP3P water model [39]. The starting model obtained from AlphaFold2.0 [23] prediction was solvated in a triclinic box with minimal distance of the model to the box wall of 1.0 nm. Chloride ions were added by replacing water molecules to neutralise the overall charge. Periodic boundary conditions were imposed in the three dimensions and the system was subjected to energy minimisation. The solvent was then equilibrated first in NVT and then in NPT ensemble for 100 ps, during which the protein atoms were restrained to the energy-minimized initial coordinates. During these stages, Berendsen thermostats were used to keep a constant temperature (300 K) and pressure (1 atm). The 500 ns production run was simulated in the NPT ensemble using the Parrinello–Rahman method to keep the pressure constant with a coupling τp = 2.0 ps. The integration time step was 0.002 ps and LINCS was the hydrogen atoms constraint algorithm used [40]. The Particle Mesh Ewald (PME) was used for the long-range interactions [41] and a cut off of 10 Å was used for the treatment of Lennard–Jones interactions. The trajectory has been analysed using VMD [42] and GROMACS routines [24]. The equilibrated part of the trajectory (100–500 ns) has been used to calculate RMSF fluctuations, hydrogen bond distances and non-bonded contacts. Hydrogen bonds are determined based on cut offs for the distance Donor–Acceptor (d < 3.3 Å) and the angle Hydrogen–Donor–Acceptor (angle < 20°). Non-bonded contacts in Table 3 are considered as present if the distance between the two carbon atoms is lower than 4.5 Å.

The interface between MB59 and CD59 was analysed by calculating an intermolecular contact’s map by the MDcons package [28] (see Supporting Information). The map of inter-residue contacts between the two chains has been calculated using 5000 frames extracted from the trajectory sampled every 100 ps. In this analysis, two residues are defined as in contact if at least two heavy atoms are within 5 Å. The map is colour coded according to the conservation of the contact during the simulation.

### 4.4. Design of Paratope-Mimicking Peptides

Based on the results of SabPred [25] and on MD results, we determined that CDR loops of MB59 that are most frequently involved in CD59 interactions are MB59, CDR1 and CDR3 loops of the V_L_ chain. Therefore, we designed three bi-cyclic peptides embedding key residues of CDR1 and CDR3 loops of the VL domain. To prevent conformational strains, the structure of the MB59 model was analysed to generate bicyclic molecules with similar conformations as the selected CDR regions observed in MB59 model. Residues lining the selected CDR portions were mutated to cysteines to allow for the usage of protocols needed to cyclise the linear peptides through the univocally determined alkylation of the thiol groups by the symmetric organo-bromide TBMB (1,3,5-Tris(bromomethyl)benzene [18].

### 4.5. Solid-Phase Peptide Synthesis and Purification

Protected amino acids and coupling agents for peptide synthesis were purchased from GL-Biochem (Shanghai, China) and IRIS Biotech GmbH (Marktrewitz, Germany). The RINK amide resin was from Novabiochem (Darmstadt, Germany). Solvents including CH_3_CN and DMF were from ROMIL (Dublin, Ireland). The analytical (Alliance e2695) and preparative (LC Prep 150) HPLCs are from Waters (Sesto, SG, Italy). LC-MS analyses were performed using an Agilent 1290 Infinity LC System coupled to an Agilent 6230 time-of-flight (TOF) MS System (Agilent Technologies, Cernusco Sul Naviglio, Italy). All certified reagents used to perform BLI analyses and OCTET R8 instruments were supplied by Sartorius (Varedo, MB, Italy).

Cyclic peptides were synthesised by solid phase synthesis following standard Fmoc chemistry protocol using a Rink-amide MBHA resin (substitution 0.56 mmol/g) and amino acid derivatives with standard side chain protections. Synthesis procedures were carried out as previously described [43,44]. After cleavage (TFA/TIS/water (90:5:5, *v*/*v*/*v*), the bicyclic peptides named pm59_sh_ (short), pm59_md_ (medium) and pm59_ln_ (long) were folded around the tribromomethylbenzene (TBMB) as previously reported for other similar molecules [7,35] and purified to homogeneity by RP-HPLC using an X-Bridge Prep C18 column (150 × 19 mm ID) applying a linear gradient of 0.1% TFA in CH3CN (solvent B) from 5% to 70% over solvent A (0.1% TFA in H_2_O) in 15 min (flow rate 15 mL/min) using a Waters LC Prep 150 HPLC system. Peptides purity and identity were assessed by LC-ESI-TOF-MS using an X-bridge C18 (5 μm, 50 × 2.1 mm ID) columns, applying linear gradients from 5 to 70% of 0.05% TFA in CH_3_CN over 0.05% TFA in H_2_O in 10 min.

### 4.6. Surface Plasmon Resonance Binding Assays

Assessment of binding kinetics and affinity between pm59 peptides and CD59 was performed at 25 °C using standard protocols on a BIACORE 3000 system (GE Healthcare, Little Chalfont, UK) with a research-grade CM5 (Carboxymethyl dextran) sensor chip and using commercial CD59 (ab134887) obtained from Abcam (Cambridge, UK). After chip preparation according to the manufacturer’s guidelines and surface activation, CD59 was immobilised onto the chip at a density of 4000 RU (0.5 mg/mL in 10 μL/min) on a NaAc buffer at pH 4.5. Bovine serum albumin (BSA) was immobilised on flow cell 2 to serve as reference. All surfaces were blocked with a 7′ injection of 1 M ethanolamine at pH 8.5.

The bicyclic acetylated CD59-targeting peptides were diluted in HBS-EP buffer (GE Healthcare, Little Chalfont, UK) and injected over the flow cells at a flow rate of 30 μL/min and concentrations ranging from 1 μM to 30 μM. Association of the complexes was allowed for 120 s and dissociation for 240 s. Regeneration of the surfaces was obtained by the sequential injection of 5 mM NaOH pulses (10 μL/min) until the established baseline was reached. Kinetics and binding data were fitted to a 1:1 stoichiometric interaction model using the global data analysis of the BIAevaluation software (GE Healthcare, Little Chalfont, UK) and Graphpad (Graphpad software, San Diego, CA, USA).

### 4.7. Biolayer Interferometry (BLI) Binding Assays

The BLI technique was used to assess the binding of the peptides towards the human recombinant CD59 protein (Abcam code ab134887). The protein was efficiently immobilized at 15 μg/mL in 10 mM NaAc at pH 4.5 on a AR2G sensor chip (Sartorius) according to the manufacturer’s instructions. An unmodified sensor chip was used as blank. The peptides were tested at increasing concentrations from 5 µM to 50 µM dissolved in Kinetic Buffer (PBS containing 0.02% Tween20, 0.1% BSA, 0.05% sodium azide) and using the same running buffer. All analyses were carried out at 25 °C; 10 mM NaOH was used to regenerate the chip surface. All mathematical manipulations were performed using the Octet Analysis Studio 12.2 software from Sartorius. Data were fitted assuming a 1:1 Langmuir binding model determining the K_D_s by the global analysis mode. Binding curves were exported and charted using GraphPad software, vers. 5.

## Figures and Tables

**Table 1:** Most frequent contacts in MD trajectory: contacts identified by the contact map (<5 Å). Selection criteria are defined in the Section 4. Persistence and distance values refer to the equilibrated part of the trajectory (100–500 ns).

Residues Involved in Intermolecular Contacts with a Conservation > 70%						
**CD59**	Y5, 8-PNPTADCK-15	31-KAGLQV-36		55-LRE-57		73-NEQ-75
**MB59**	Y31, 33-SNNK-36, Y38, A40 (V_L_ CDR1)	L52, 55-YW-56 (V_L_ CDR2)	97-YYSTP-101 (V_L_ CDR3)	T162, 164-SSY-166 (V_H_ CDR1)	Y184, 186-SSS-188 (V_H_ CDR2)	R231, 233-PGMD-236 (V_H_ CDR3)

**Table 2:** Most frequent contacts in MD trajectory: H-bonding.

H-Bonding Interactions					
MB59 Residue (Atom)	CDR	CD59 Residue (Atom)	Percentage of Persistence (%)	Average Distance (Sigma) (Å) *	Type of Interaction
N34 (ND2)	V_L_ CDR1	N73 (O)	93.0	2.8 (0.1)	Stable H-bond
Y97 (OH)	V_L_ CDR3	L34 (O)	52.1	2.9 (0.2)	Stable H-bond
S33 (OG)	V_L_ CDR1	N73 (ND2)	52.0	3.7 (1.2)	Transient H-bond
S165 (OG)	V_H_ CDR1	E57 (OE2)	56.0	5.0 (2.8)	Transient H-bond
T162 (OG1)	V_H_ CDR1	R56 (O)	42.4	3.8 (1.5)	Transient H-bond
S164 (OG)	V_H_ CDR1	L55 (O)	32.6	4.8 (2.4)	Transient H-bond

* Standard deviation of distances in the trajectory structures (sigma) is reported in parenthesis.

**Table 3:** Most frequent contacts in MD trajectory: non-bonding interactions.

Non Bonded Interactions Involving V_L_ CDR Regions				
MB59 Residue (Atom)	CDR	CD59 Residue (Atom)	Percentage of Persistence (%)	Average Distance (Sigma) (Å) *
S33 (CB)	V_L_ CDR1	Y5 (CE1)	96.2	3.7 (0.4)
N34 (CB)	V_L_ CDR1	V36 (CG2)	33.5	4.8 (0.6)
Y38 (CE1)	V_L_ CDR1	A12 (CB)	85.8	4.2 (0.3)
Y38 (CE1)	V_L_ CDR1	Q35 (CB)	82.2	4.2 (0.4)
A40 (CB)	V_L_ CDR1	L34 (CD1)	57.2	4.5 (0.6)
Y97 (CE2)	V_L_ CDR3	L34 (CB)	78.3	4.2 (0.3)
Y97 (CE2)	V_L_ CDR3	Q35 (CG)	67.2	4.3 (0.6)
Y98 (CD2)	V_L_ CDR3	A12 (CB)	96.4	3.8 (0.3)
T100 (CG2)	V_L_ CDR3	T11 (CB)	45.5	4.9 (0.9)
L52 (CD2)	V_L_ CDR2	L34 (CD2)	93.1	4.0 (0.4)
L55 (CB)	V_L_ CDR2	L34 (CD2)	95.1	3.9 (0.4)
W56 (CH2)	V_L_ CDR2	K31 (CD)	64.4	4.5 (0.7)

* Standard deviation of distances in the trajectory structures (sigma) is reported in parenthesis.

**Table 4:** Sequences of bicyclic paratope-mimicking peptides (pm59). Sequences include key residues of CDR1 and CDR3 loops of MB59 V_L_ chain in short (sh), medium (md) and long (ln) versions. Bi-cyclisation was achieved using TBMB that connected the cysteines (bold) to form bicyclic molecules. The calculated (monoisotopic) and experimental MWs are also reported.

Synthesised Peptides	Sequence	Calculated MW (amu)	Experimental MW (amu)
pm59_sh_	Ac-CNKYLCQYYSTC-NH_2_	1642.66	1642.66
pm59_md_	Ac-CNNKYLCQYYSTPC-NH_2_	1855.03	1854.76
pm59_ln_	Ac-CNNKYLACQQYYSTPC-NH_2_	2054.24	2053.85

**Table 5:** SPR kinetics parameters related to binding curve analyses between pm59 peptides and CD59.

**(a) pm59_sh_**					
**Conc. (μM)**	**K_D_ (M)**	**ka (1/Ms)**	**kd (1/s)**	**Chi2**	**SE (RI)**
5	4.69 × 10^−7^	7.26 × 10^3^	3.40 × 10^−3^	0.0505	0.0505
7.5	1.24 × 10^−6^	4.21 × 10^3^	5.23 × 10^−3^	0.0837	0.0837
10	1.64 × 10^−6^	3.01 × 10^3^	4.93 × 10^−3^	0.1040	0.1300
15	2.39 × 10^−6^	2.05 × 10^3^	4.90 × 10^−3^	0.1620	0.1650
20	2.61 × 10^−6^	1.56 × 10^3^	4.08 × 10^−3^	0.1160	0.1390
25	4.13 × 10^−6^	1.16 × 10^3^	4.81 × 10^−3^	0.0370	0.0770
30	4.46 × 10^−6^	1.23 × 10^3^	5.48 × 10^−3^	0.2080	0.1390
Average	2.42 × 10^−6^	2.93 × 10^3^	4.69 × 10^−3^	0.1090	0.1190
**(b) pm59_md_**					
**Conc. (μM)**	**K_D_ (M)**	**ka (1/Ms)**	**kd (1/s)**	**Chi2**	**SE (RI)**
2.5	1.38 × 10^−6^	5.96 × 10^3^	8.14 × 10^−3^	0.0173	0.0405
5	9.02 × 10^−7^	7.08 × 10^3^	6.38 × 10^−3^	0.0199	0.0592
10	1.09 × 10^−6^	4.83 × 10^3^	5.26 × 10^−3^	0.0427	0.0959
20	3.10 × 10^−6^	2.29 × 10^3^	7.10 × 10^−3^	0.0901	0.1380
30	4.39 × 10^−6^	1.27 × 10^3^	6.27 × 10^−3^	0.0383	0.0846
40	3.88 × 10^−6^	1.32 × 10^3^	5.111 × 10^−3^	0.0995	0.1500
50	4.90 × 10^−6^	0.98 × 10^3^	4.80 × 10^−3^	0.1301	0.1670
average	3.13 × 10^−6^	2.93 × 10^3^	5.82 × 10^−3^	0.0625	0.1158

**Table 6:** BLI kinetics parameters related to binding curve analyses between pm59 peptides and CD59.

**(a) pm59_sh_**								
**Conc. (μM)**	**K_D_ (M)**	**KD Error**	**ka (1/Ms)**	**ka Error**	**kdis (1/s)**	**kdis Error**	**Full X^2^**	**Full R^2^**
5	8.646 × 10^−7^	2.522 × 10^−8^	1.133 × 10^3^	2.252 × 10^1^	9.798 × 10^−4^	2.092 × 10^−5^	0.0132	0.9793
10	4.983 × 10^−6^	8.369 × 10^−8^	5.236 × 10^2^	0.814 × 10^1^	2.609 × 10^−3^	1.647 × 10^−5^	0.0269	0.9888
20	9.921 × 10^−6^	1.726 × 10^−7^	2.939 × 10^2^	0.473 × 10^1^	2.916 × 10^−3^	1.910 × 10^−5^	0.0653	0.9852
30	8.446 × 10^−6^	1.428 × 10^−7^	4.510 × 10^2^	0.658 × 10^1^	3.809 × 10^−3^	3.241 × 10^−5^	0.2877	0.9524
40	1.496 × 10^−5^	2.407 × 10^−7^	1.966 × 10^2^	0.281 × 10^1^	2.941 × 10^−3^	2.150 × 10^−5^	0.2194	0.9793
50	1.59 × 10^−5^	2.521 × 10^−7^	1.779 × 10^2^	0.244 × 10^1^	2.832 × 10^−3^	2.244 × 10^−5^	0.4885	0.9760
average	9.182 × 10^−6^	1.529 × 10^−7^	4.627 × 10^2^	0.786 × 10^1^	2.681 × 10^−3^	2.214 × 10^−5^	0.1835	0.9768
**(b) pm59_md_**								
**Conc. (** **μ** **M)**	**K_D_ (M)**	**KD Error**	**ka (1/Ms)**	**ka Error**	**kdis (1/s)**	**kdis Error**	**Full** **X^2^**	**Full R** **^2^**
5	2.080 × 10^−10^	3.040 × 10^−7^	9.630 × 10^2^	3.690 × 10^1^	2.003 × 10^−7^	6.312 × 10^−7^	0.0096	0.9405
10	1.228 × 10^−6^	2.150 × 10^−7^	1.562 × 10^3^	1.801 × 10^1^	1.919 × 10^−3^	2.527 × 10^−5^	0.0170	0.9521
20	4.124 × 10^−6^	7.747 × 10^−7^	8.889 × 10^2^	1.383 × 10^1^	3.666 × 10^−3^	3.857 × 10^−5^	0.1905	0.8989
25	6.517 × 10^−6^	1.474 × 10^−7^	7.833 × 10^2^	1.561 × 10^1^	5.105 × 10^−3^	5.452 × 10^−5^	0.0690	0.9216
30	6.094 × 10^−6^	1.362 × 10^−7^	7.497 × 10^2^	1.435 × 10^1^	4.569 × 10^−3^	5.26 × 10^−5^	0.3972	0.8823
40	8.381 × 10^−6^	1.896 × 10^−7^	5.642 × 10^2^	1.101 × 10^1^	4.728 × 10^−3^	5.413 × 10^−5^	0.7459	0.8823
average	4.391 × 10^−6^	1.460 × 10^−7^	9.186 × 10^2^	1.829 × 10^1^	3.331 × 10^−3^	4.804 × 10^−5^	0.2839	0.9130

## Data Availability

Data are available from the authors.

## Supplementary Materials

The supporting information can be downloaded at: https://www.mdpi.com/article/10.3390/ijms24108561/s1.

## Abbreviations

PDB, Protein Databank; MD, molecular dynamics; AI, artificial intelligence; AF, AlphaFold; RMSD, root means square deviation; RMSF, root mean square fluctuation; pm59, paratope-mimicking peptides targeting CD59; MB59, single chain variable fragment scFv targeting CD59; NVT, isothermal-isovolume; NPT, isobaric-isothermal.
