# Supplementary material for: Unveiling CD59-Antibody Interactions to Design Paratope-Mimicking Peptides for Complement Modulation
Source: Int J Mol Sci. 2023 May 10;24(10):8561. doi: 10.3390/ijms24108561 (PMC10218266; doi:10.3390/ijms24108561)

Figure S1. Map of inter-residue contacts between MB59 and CD59 chains generated using the MDcons package along the trajectory sampled every 100ps. Two residues are considered to be in contact if at least two heavy atoms are within 5 Å. The map is color coded considering the conservation of the contact during the simulation.

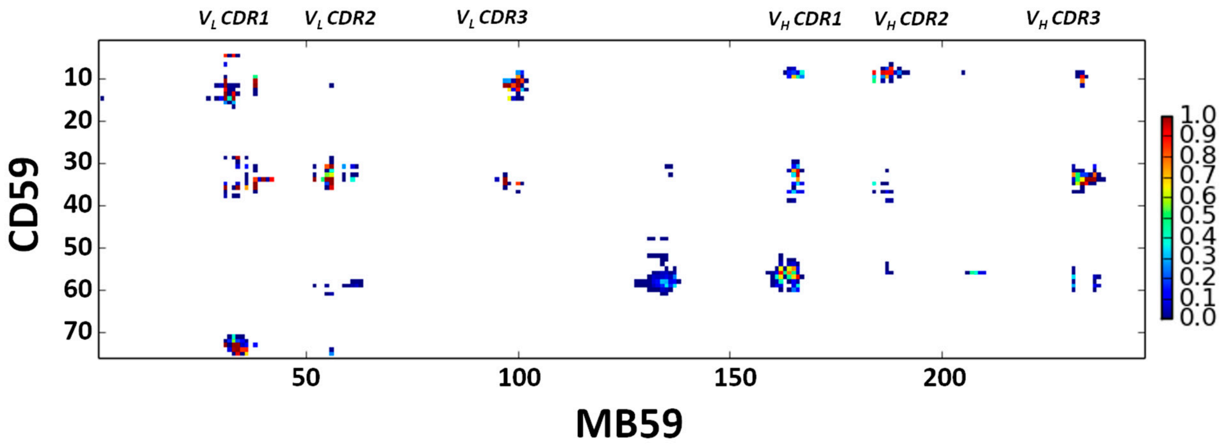

Figure S2. Time evolution of non-bonded contacts in CDR1 (**A**) and CDR3 (**B**) of MB59 V_L chain.

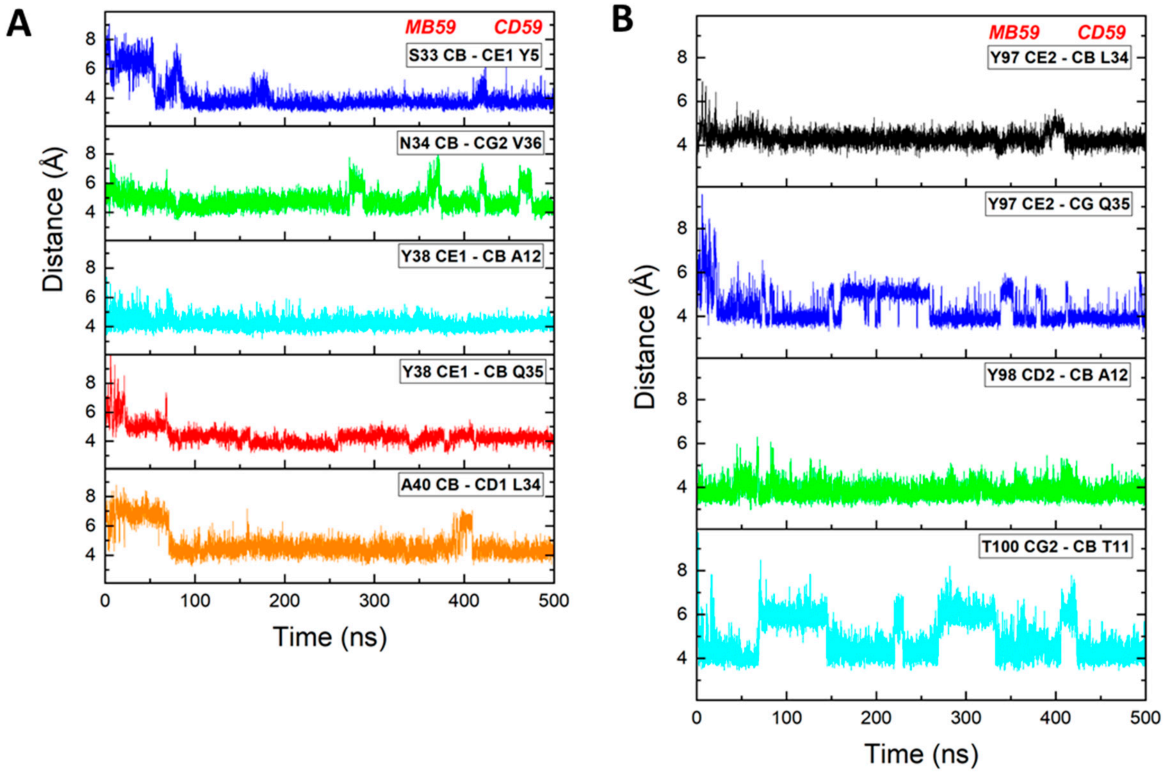

Figure S3. LC-ESI-TOF-MS analysis of the byciclic pm59$_{sh}$ peptide including RP-HPLC profile (top) and mass spectrum (bottom).

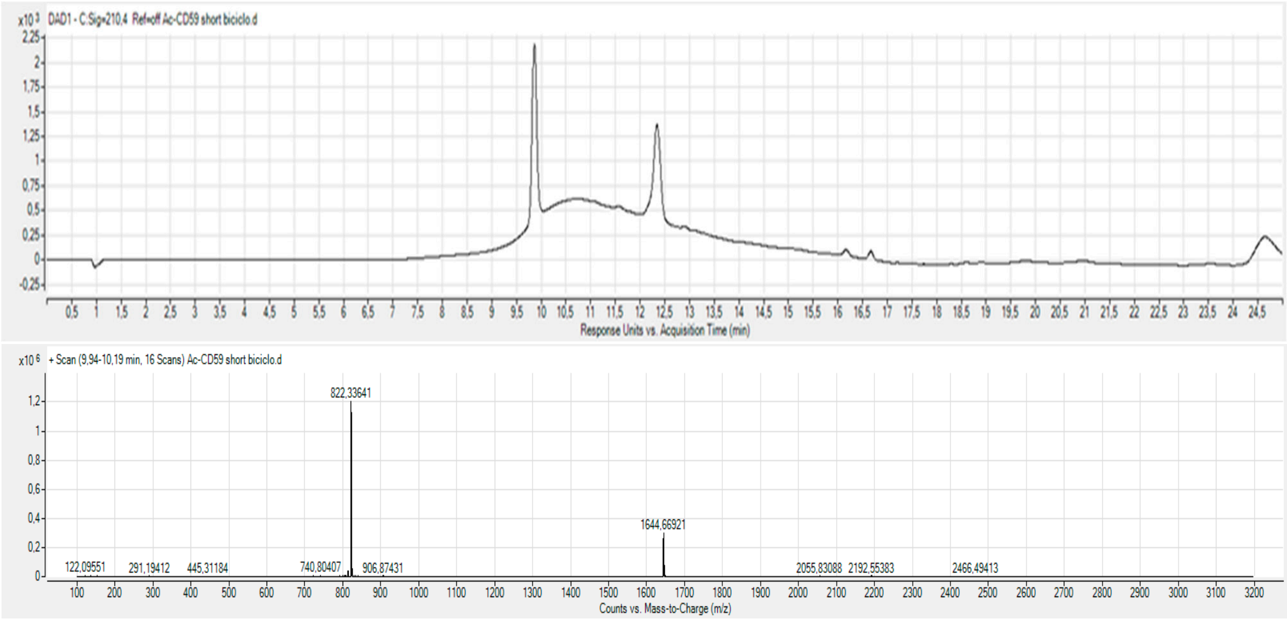

Figure S4. LC-ESI-TOF-MS analysis of the byciclic pm59$_{md}$ peptide including RP-HPLC profile (top) and mass spectrum (bottom).

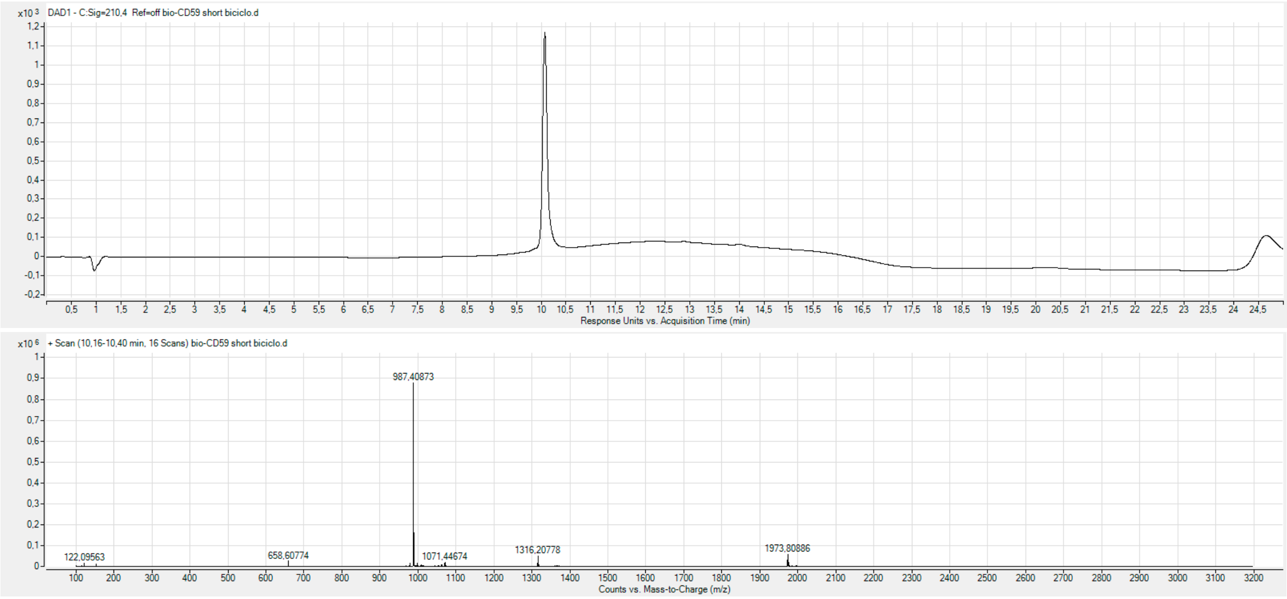

Figure S5. LC-ESI-TOF-MS analysis of the byciclic pm59$_{ln}$ peptide including RP-HPLC profile (top) and mass spectrum (bottom).

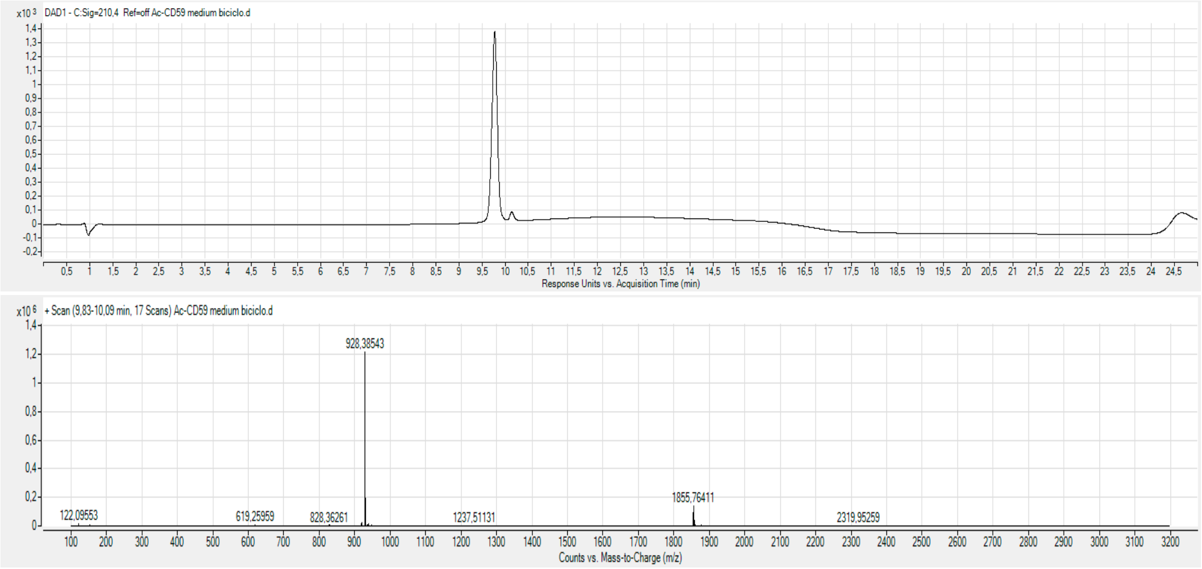

Supplement: Supplementary file 1 [file ijms-24-08561-s001.zip › ijms-2360221-supplementary.pdf]
